# Soft Sensors in the Primary Aluminum Production Process Based on Neural Networks Using Clustering Methods

**DOI:** 10.3390/s19235255

**Published:** 2019-11-29

**Authors:** Alan Marcel Fernandes de Souza, Fábio Mendes Soares, Marcos Antonio Gomes de Castro, Nilton Freixo Nagem, Afonso Henrique de Jesus Bitencourt, Carolina de Mattos Affonso, Roberto Célio Limão de Oliveira

**Affiliations:** 1 Institute of Technology, University of Pará, Belém 66075-110, Brazil; fms@ufpa.br (F.M.S.); carolina@ufpa.br (C.d.M.A.); limao@ufpa.br (R.C.L.d.O.); 2 Department of Automation, Specialist engineer, Aluminum of Brazil (ALBRAS), Barcarena 68445-000, Brazil; marcos.castro@albras.net; 3 Reduction Area, Process Engineering Manager, Aluminum of Brazil (ALBRAS), Barcarena 68445-000, Brazil; nilton.nagem@albras.net; 4 Department of Automation, Manager of Energy, Utilities, Automation, and Predictive, Aluminum of Brazil (ALBRAS), Barcarena 68445-000, Brazil; afonso.bitencourt@albras.net

**Keywords:** primary aluminum production, soft sensor, neural network, real data, estimation, clustering methods

## Abstract

Primary aluminum production is an uninterrupted and complex process that must operate in a closed loop, hindering possibilities for experiments to improve production. In this sense, it is important to have ways to simulate this process computationally without acting directly on the plant, since such direct intervention could be dangerous, expensive, and time-consuming. This problem is addressed in this paper by combining real data, the artificial neural network technique, and clustering methods to create soft sensors to estimate the temperature, the aluminum fluoride percentage in the electrolytic bath, and the level of metal of aluminum reduction cells (pots). An innovative strategy is used to split the entire dataset by section and lifespan of pots with automatic clustering for soft sensors. The soft sensors created by this methodology have small estimation mean squared error with high generalization power. Results demonstrate the effectiveness and feasibility of the proposed approach to soft sensors in the aluminum industry that may improve process control and save resources.

## 1. Introduction

Although pure aluminum (Al) is one of nature’s most abundant elements, it is extremely difficult to extract, and extraction is not possible without the occurrence of some chemical reaction. Al is always attached to some other chemical element in the form of salts or oxides, which makes separation necessary. In the 1880s, the young students Charles Hall and Paul Héroult used electrolysis to separate the Al of oxygen from alumina (Al_2_O_3_) grains into salts fluxes such as cryolite (Na_3_AlF_6_). This is the Hall–Héroult process [1,2] by which the primary aluminum industries perform can obtain Al up to 99.9% purity. Basically, this is the separation of alumina into alumina and oxygen, but the process also requires the participation of other elements such as flux salts, gases, and chemical additives to maintain process stability, which makes the process more complex [1,3].

For complex industrial processes, mathematical modeling is also a complex task, in such a way that representing a process in a completely analytical way becomes impracticable. The use of approximate and hybrid representations produces very satisfactory results, although they are not scalable from a certain point [4]. As the scientific improvement of modeling and identification techniques [5], this task has been dealt with more easily and in various areas of knowledge, although the great difficulty of performing dynamic modeling of nonlinear processes remains.

This process of modeling and identification of dynamic nonlinear systems has advanced considerably with the use of artificial intelligence and machine learning techniques, which have been applied in the last few decades with excellent results [6,7,8,9,10]. The success of using these “intelligent” paradigms in modeling dynamic systems is due to the little knowledge required to perform modeling (only a reasonable amount of data is required) compared to other forms of analytical modeling, and also because they are naturally nonlinear models. Among these “intelligent” techniques used for nonlinear dynamic modeling [11,12], one of the most used is artificial neural networks. The use of artificial intelligence in dynamic modeling based on data is sometimes referred to as soft sensors.

Soft sensors are computationally implemented, data-driven models that provide online estimates of process variables that cannot be continuously and/or reliably measured online for technological and/or economic reasons [4,13]. These techniques use process variables that are measured and recorded reliably online using available physical sensors or offline through laboratory analysis results.

Data-driven soft sensors have wide success in the industry, because of its practicability, robustness, and flexibility to be developed and applied to a wide range of processes, in addition to their independence from a process mathematical model [14,15]. There are a number of methods for implementing flexible data-driven sensors for industrial processes. Some of the most commonly used linear methods are multi-statistic regression algorithms, such as principal component analysis (PCA) [16,17,18,19] and partial least squares (PLS) [20,21,22,23]. These methods have more practical applications because of their simplicity and can work with some invariance in time; however, they have some disadvantages because they are prone to errors in the presence of data impurities (missing values and outliers) and are inadequate to deal with nonlinearities.

Nonlinear processes are usually modeled with nonlinear structures such as artificial neural networks (ANN) [24,25,26,27,28], neuro-fuzzy [29,30,31], Gaussian process regression support vectors [32,33,34], and support vector machines [35,36,37]. The most common types of ANN are multi-layer perceptron (MLP) and radial basis function networks (RBFN). The literature has shown that ANN is especially suitable for implementation of soft sensors, and these have indeed been used [38,39,40,41,42,43,44,45,46,47]. More recently, deep learning has been used to create soft sensors also successfully [48,49,50,51].

Due to the complexity of the primary aluminum production process, it is interesting to use data-driven soft sensors to measure the most important variables of this process, since it is a nonlinear, time-variant, and distributed-parameter dynamic process. Moreover, since the electrolytic process of oxidized alumina reduction is very aggressive, it is not possible to have temperature measurements in real time, since the chemical bath corrodes the thermocouple (usually a thermocouple can do 50 measurements every 24 h).

ANNs have been used as a powerful artificial intelligence technique to construct models based on data in the Al industry [52,53,54,55]. In this way, ANNs are also widely used to implement soft sensors. In the Al smelting process, ANN has been used in a minor way to simulate and model processes [56,57,58], while in parallel other techniques like clustering help to identify pots with common behaviors to enhance the knowledge derived from the data [59]. In major part, mathematical techniques have been used to create models to emulate the Al production process [60,61,62,63,64].

An industrial Al plant has hundreds of pots working simultaneously, so this feature contributes to make the production process more complex as a whole, often requiring many human interventions [3]. Methodologically, it is possible to apply neural modeling in one of the following approaches:

- A single ANN for all electrolysis pots; in this approach, the results are barely satisfactory, since it is very difficult for ANN to capture the behavioral differences of all pots.
- An ANN for each pot, which might be too complex and difficult to apply, since it is necessary to tune hundreds of ANNs.
- One ANN for a certain cluster of pots, which present similar behaviors.

**Figure 1:**
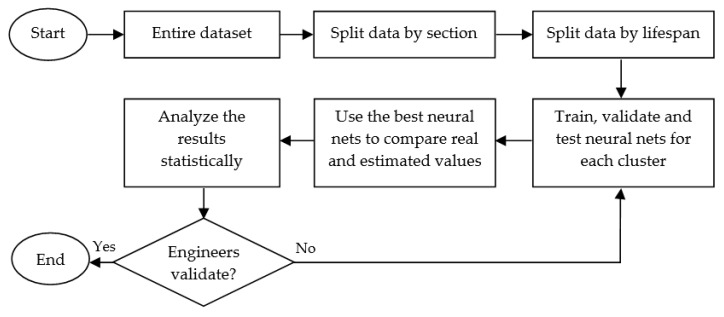
Flowchart of the proposed method.

This paper describes the process of designing soft sensors using the third methodology, which could present the best trade-off between complexity and quality of results. The engineering expertise is useful for determining the key process variables to include, and the ANN technique helps in variable indirect estimation within electrolytic bath furnace modeling using real data from an Al smelter plant. This paper’s major contributions are as follows: clustering data by pots section; considering three different phases of pots, based on lifespan division; and comparing and proposing neural network estimators as soft sensors to replace manual measurements with automatic. The results show this is possible, since the models generate estimations with small errors. It is important to highlight ANN models created are dynamic, because delayed inputs were considered to estimate the current outputs. Briefly, the flowchart of the proposed method is presented by Figure 1.

The rest of this work is organized as follows. Section 2 describes the primary Al production process and describes the layout of the Al smelter concerned in this paper. Section 3 addresses in detail the design of the ANN-based estimation models. Results and discussions are presented in Section 4. Finally, Section 5 provides the conclusions.

## 2. Brief Description of the Primary Aluminum Production Process

Softness, lightness, high thermal conductivity, and high recyclability are important properties of Al. A wide variety of products are derived from this metal, which has helped it to become the most frequently consumed nonferrous metal around the world [64]. The primary Al production process is complex, due to the handling of variables from multiple disciplines, such as electrical, chemical, and physical [65].

The raw material of Al is alumina. Direct Al extraction from alumina requires a temperature over 2000 °C [66]. The machinery to maintain this high temperature is expensive, and so is the energy waste under these requirements. From the late nineteenth century, the Hall–Héroult process has been used as an alternative to produce Al, as it consumes less energy and requires a lower temperature (about 960 °C) [1,2,3]. To reduce the heat, cryolite is used as an electrolytic bath and several chemical components are added together with alumina [67].

**Figure 2:**
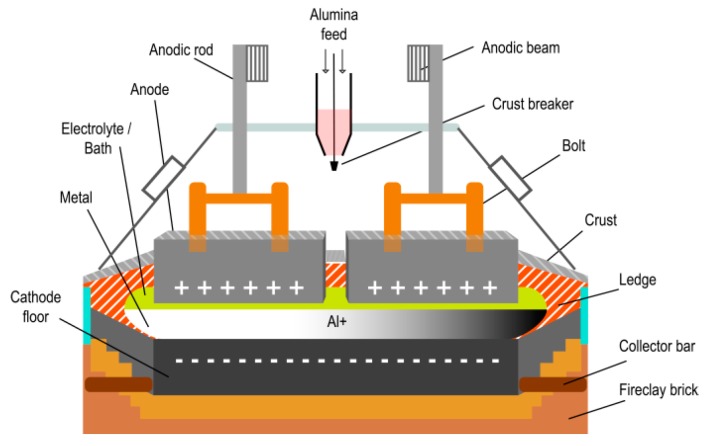
Example of a pot and its parts.

This process is widely known as Al smelting, which uses electrolysis pots, also named pots or reduction pots [68]. A pot (Figure 2) consists of a steel shell with a lining of fireclay brick for heat insulation, which, in turn, is lined with carbon bricks to hold the molten electrolyte. Steel bars carry the electric current through the insulating bricks into the carbon cathode floor of the pot. Carbon anode blocks are hooked onto steel rods and immersed in the electrolyte. Alumina molecules are dissolved by the heat and decomposed into Al and oxygen (O) by electric current that flows through the electrolyte [69]. In modern smelters, process-control computers connected to remote sensors ensure optimal operation of electrolysis pots [70]. Electrolysis furnaces are organized within reduction rooms—standard Al smelting uses around four reduction rooms and between 900 and 1200 pots in total, depending on the smelter.

According to the stoichiometric relation (Equation (1)), alumina is consumed in the production process together with the solid carbon of the anodes. Theoretically, this consumption is 1.89 kg of Al_2_O_3_ for each 1.00 kg of Al^+^, whereas 0.33 kg of carbon (C^+^) produces 1.22 kg of carbon dioxide (CO_2_). In practice, typical values are 1.93 kg Al_2_O_3_ to 1.00 kg Al^+^ and between 0.40 and 0.45 kg of C^+^ to 1.00 kg Al^+^, with an emission of about 1.50 kg CO_2_ [69].

2Al_2_O_3_ (dissolved) + 3C+ (solid) => 4Al+ (liquid) + 3CO_2_ (gas). (1)

**Figure 3:**
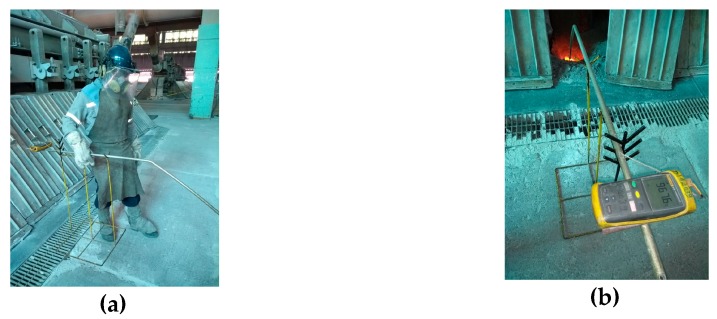
Pot temperature measurement: (**a**) human operator; and (**b**) thermocouple connected to display the temperature value.

Several sensors monitor the entire process continuously, acquiring data from the entire plant. Data are stored and organized in databases, which became a rich patrimony of the plants, as they keep the historical information on each production pot. This data collection supports the building of automatic decision-making systems and guides for the engineers [71,72,73,74]. Many control systems display the data acquired in real time for the permanent monitoring of the process. Plant control systems for Al smelting have two modes of operation [74,75]:

- Automatic control: Data are collected and processed by computers and/or microcontrollers, which then drive a control action on the plant without direct human intervention. Examples: control of electrical resistance of the pot by the anode–cathode distance (ACD) using pulse width modulation (PWM) to drive the lifting/lowering of anodes; and the control of alumina to be added to the electrolytic bath through mathematical models.
- Manual control: Data are collected through plant floor sensors or manually measured by process operators, but the calculation of the output is performed by the process engineers, taking into account mathematical models and their expertise. Examples: thermocouple to measure the temperature of the pots (Figure 3), percentage of fluoride alumina in the bath (laboratory result), metal level of the pot, replacement of anodes, and Al tapping from the pot.

**Figure 4:**
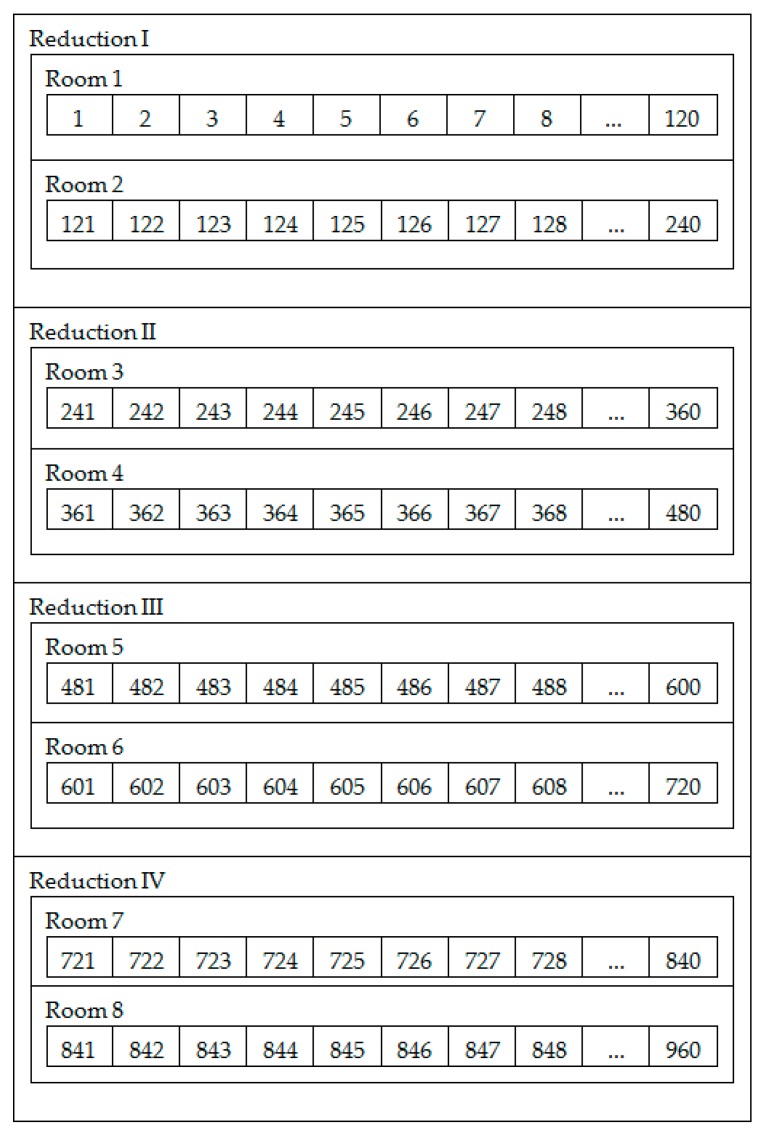
Overall layout of the smelter made up of four reductions, eight rooms, and 960 pots.

The experiments conducted in this paper were derived from a real Brazilian Al smelter, from which real data were used to generate results. The pots are arranged in four reductions, each of which has two rooms, and each room has 120 pots, resulting in 960 pots. Figure 4 shows the overall layout of this factory.

**Figure 5:**
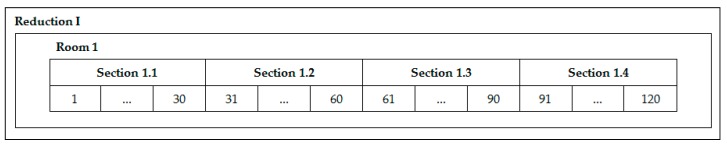
Section layout by room.

Electrically, Al reduction pots are connected in series. This connection allows the continuous electric current (approximately 180 kA) to be the same in all pots. It should be noted that for a room there are two lines of electricity, each line composed of two sections, which in turn contain 30 pots, resulting in 32 different sections for the entire smelter. Figure 5 outlines the arrangement of the sections for reduction I and the first room. This same organization is present in all rooms of the smelter concerned and these pots’ disposition was used as clusters empirically; each cluster is a section.

## 3. Design of Estimation Models

The full database has hundreds of thousands of samples and hundreds of process features (variables) from 2006 to 2016. The following subsection depicts the preprocessing steps performed in the original database in order to generate the datasets used in this work.

### 3.1. Data Extraction, Imputation, and Split

Data extraction considered the entire life of each pot, in other words a lifespan from 1 to 1500 days, taking into account an average of five years of operation. Table 1 shows all variables available in the database. Therefore, features selection considered Pearson correlation (R), between input and output, to rank variables by degree of importance. It is important to know that some variables have a large number of null values, so they were discarded. R is calculated as:

(2) $$R_{xy}=\frac{\sum_{i=1}^{n}\left(x_{i}-\bar{x}\right)\left(y_{i}-\bar{y}\right)}{\sqrt{\sum_{i=1}^{n}{\left(x_{i}-\bar{x}\right)}^{2}}\sqrt{\sum_{i=1}^{n}{\left(y_{i}-\bar{y}\right)}^{2}}},$$

where *n* is sample size, *x_i_* and *y_i_* are the individual sample points indexed with *i*, and $\bar{x}$ and $\bar{y}$ are the sample averages.

Table 2 lists the most important inputs associated with output variables selected to create the estimation models. Firstly, the inputs have been determined after a Pearson correlation study (Equation (2)). After that, process engineers validated the feature selection to the model. It is important to note that all input variables are delayed by one step, because neural models emulate a first order dynamic system with delayed inputs to estimate the current output. The final selected dataset had about 1,728,000 samples and eleven inputs and three outputs.

**Figure 6:**
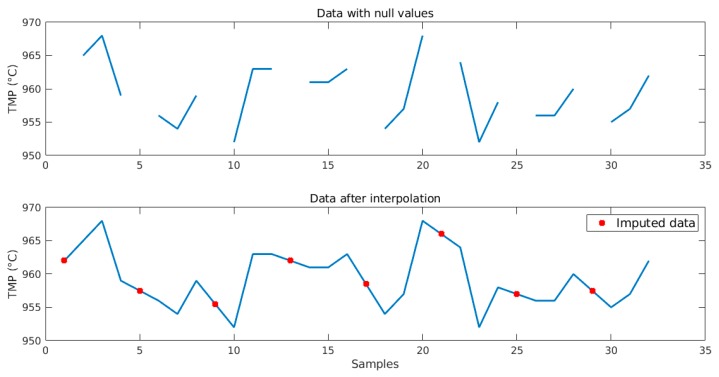
Example of data imputation for bath temperature.

Some variables, such as temperature, percentage of fluoride, and metal level, are collected manually by physical sensors or through laboratory analysis, generating different sampling frequencies. Other variables, for instance real resistance and raw voltage, are collected online via sensors without human interference. Most of the variables are sampled on a daily basis; however, variables that are collected manually have other sampling frequencies. This fact causes null data to be present between measurements when combining variables from different samplings. Missing data were imputed by calculating a linear interpolation between the previous and subsequent measurements, according to the variable sampling. According to process engineers, linear interpolation fits well, because the chemical process is slow and it has been validated before. Figure 6 shows an imputation example for bath temperature. The soft sensors described in this work have the advantage of being capable of estimating missing data after they have been properly trained.

**Figure 7:**
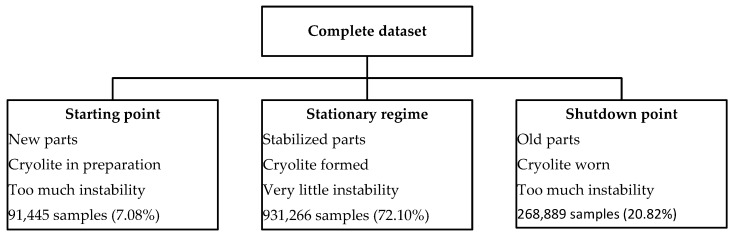
Description of each lifespan division.

Process engineers also agree there are three different types of behaviors produced by pots according to their lifespan: a lifespan of 1–100 days is considered a “starting point”; 101–1200 days as a “stationary regime”; and 1201–1500 days as the “shutdown point”. This lifespan division is the second method used to cluster the entire dataset (the first is clustering by section, explained before). These ranges may vary according to the pot, but they are the same on average. Figure 7 summarizes behaviors and the amount of data for each lifespan division.

**Figure 8:**
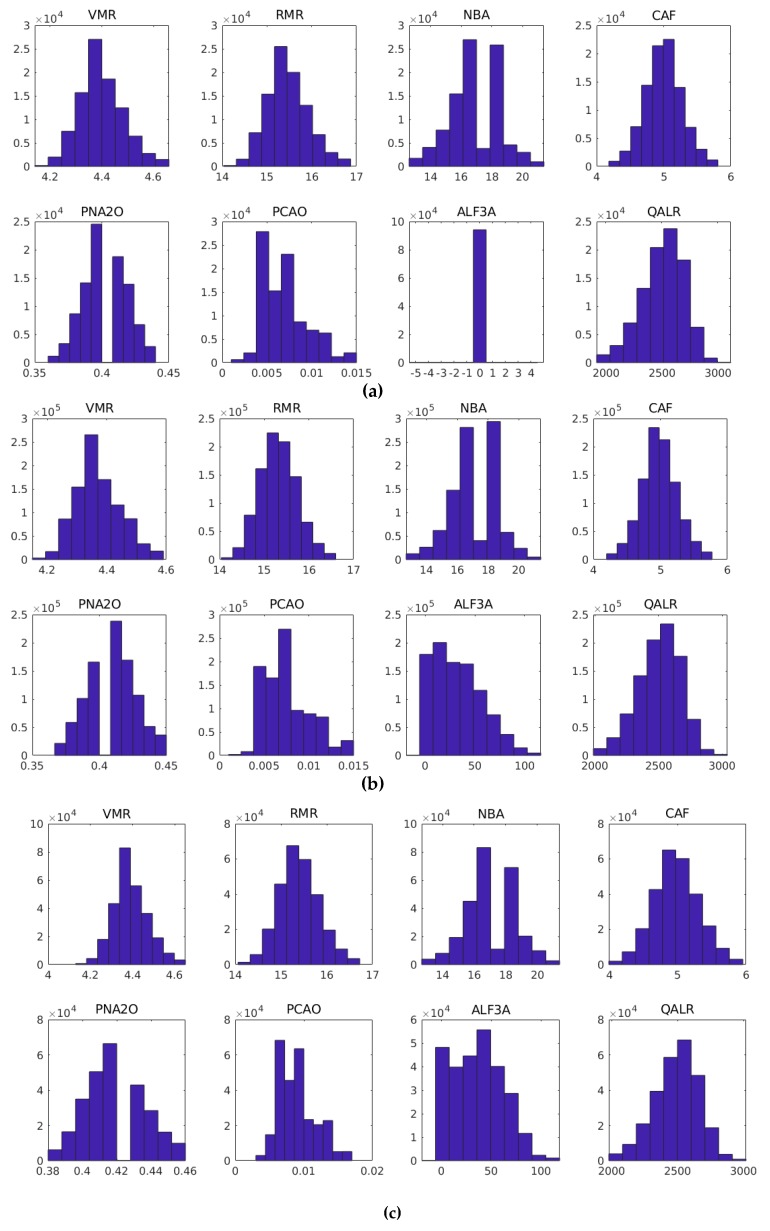
Input variables histogram: (**a**) starting point; (**b**) stationary regime; and (**c**) shutdown regime.

The different behaviors also may be verified when the dataset of each group is statistically analyzed. Figure 8 shows histograms of each input variable for each group. The ALF3A variable has zero values at the starting point, because it is not observed in this phase, so this variable may be discarded when models for this phase are created. The PNA2O variable at the starting point has a larger number of samples less than 0.4; in the stationary regime and shutdown point, the higher concentration of samples is more than 0.4. The behavior of input variables between stationary regime and shutdown point is similar.

**Figure 9:**
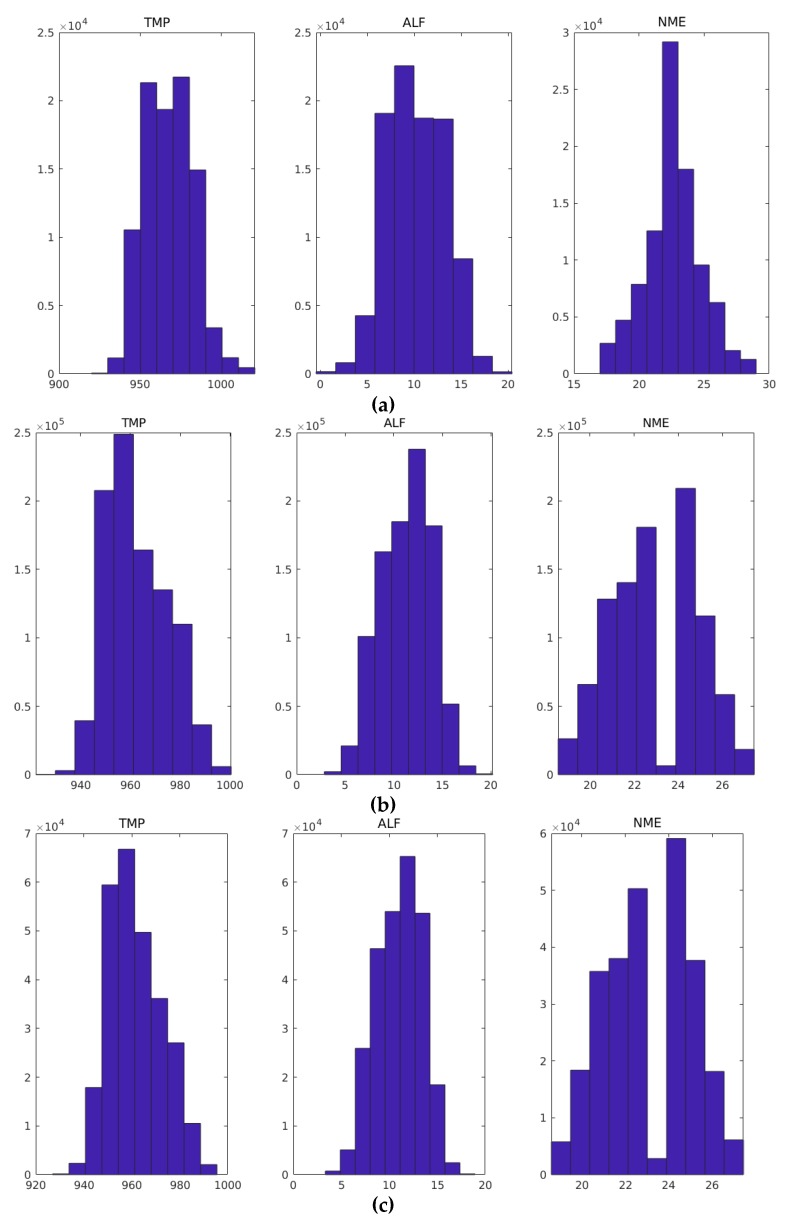
Output variables histogram: (**a**) starting point; (**b**) stationary regime; and (**c**) shutdown point.

Analyzing the output variables histogram for each behavior (Figure 9), it is possible to observe that the TMP variable at the starting and shutdown points had a range of values greater than the stationary regime, ratifying the instability thesis. Another behavior verified was about the NME variable: at the starting point it had a large accumulation of samples at 24, but in the stationary and shutdown phases the accumulation was 25. The ALF variable at the starting point had a larger sample concentration less than 10; in the other two phases the concentration was greater than 10.

**Figure 10:**
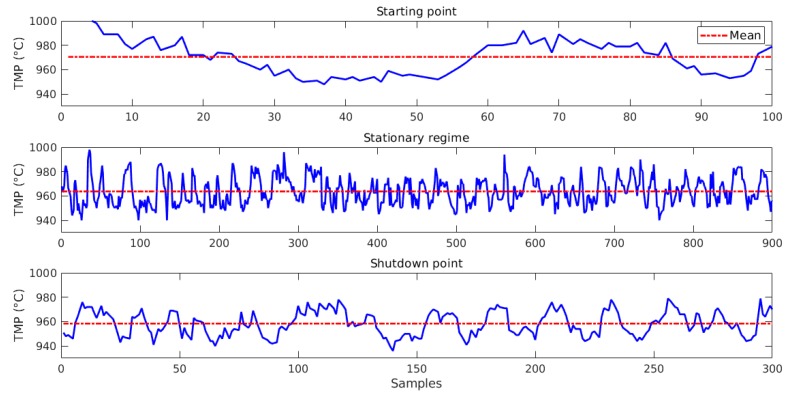
Bath temperature variation of the pot 5.

Besides histograms, the difference in TMP variation can be observed in the three phases by Figure 10. In starting point, the mean is equals 970.5 °C, because the pot must be reheated; in stationary regime, the mean decreases to 963.7 °C, the standard mean of the plant; and in shutdown point, it also decreases to 958.8 °C, since the pot is being cooled to turn off. TMP was chosen to perform this analysis, because it is one of the most monitored process variables.

The following subsection shows the steps performed in the original database in order to generate the resulting models.

### 3.2. Strategy for Modeling

Data clustered by each section and by each lifespan division were used to build models to estimate TMP, ALF, and NME using the ANN technique. It is important to know that each ANN model has only one of three outputs and two different training algorithms were used to create them: Levenberg−Marquardt (LM) and back propagation (BP). Besides, three strategies were used for each technique:

1. Consider 70% of the data from each cluster to train, 15% to validate, and 15% to test the models.
2. Consider data from all pots of one entire section to train the models, except for one pot of the respective section to test the model. This was applied to section clustering and lifespan division.
3. Dataset standardization was done using the z-score method.

The z-score generates a standardized dataset with average equal to 0 and standard deviation equal to 1 and it is expressed by:

(3) $$z=\frac{x-\mu }{\sigma },$$

where *x* is the value to be standardized, μ is the average of the variable, and σ is the standard deviation of the variable.

Table 3 shows the division of the complete dataset for the modeling process: for each lifespan division or all datasets and two different learning algorithms. Moreover, three strategies were used for each technique, 32 different pot sections, whole dataset, and three outputs, resulting in 594 different models, initially.

Each model was trained ten times, because the initial weights of the neural network and the division of training and validation data are random, according to a Gaussian probability density function. In total, 5760 neural networks were created considering clustered data, whereas 2880 models use the LM algorithm and 2880 use the BP algorithm. The pseudocode (Algorithm 1) summarizes the entire modeling process.
**Algorithm 1.** Pseudocode for modeling process using clustered dataset.EXPERIMENTS = 10; TOTAL_POTS = 960; POTS_BY_SECTION = 30; TOTAL_OUTPUTS = 3; **for** i_exp = 1 **to** EXPERIMENTS **do** **for** i_out = 1 **to** TOTAL_OUTPUTS **do**  **for** i_pot = 1 **to** 30 **to** TOTAL_POTS **do**   a) Get data from a section:     (index_pot >= i_pot and index_pot <= (i_pot + POTS_BY_SECTION − 1).   b) Create input and output (i_out) data matrices.   c) Split data between training and validation datasets.   d) Define parameters of the ANN model.   e) Create ANN model.   f) Train ANN model.   **for** i_test = i_pot **to** (i_pot + POTS_BY_SECTION − 1) **do**    g) Get data by index_pot = i_test.    h) Create input and output (i_out) data matrices.    i) Simulate ANN model using data by (step h)).    j) Calculate and store MSE and R values.    k) Check if MSE and R values are better than previous model. If true, store model.   **end_for**
  **end_for**
 **end_for** **end_for** **print/plot** MSE_test_ values by each experiments and output variable **print/plot** R_test_ values by each experiments and output variable l) Calculate MSE_test_ and R_test_ average: **print** MSE_global_ by each output variable **print** R_global_ by each output variable


The mean squared error (MSE) and the R between target and estimated values were considered as quality metrics of the models. MSE is defined as:

(4) $$MSE=\frac{1}{n}\sum_{i=1}^{n}{\left(y_{i}-{\hat{y}}_{i}\right)}^{2},$$

where *n* is the number of samples, and *y_i_* and ${\hat{y}}_{i}$ are the target and estimated values by the model, respectively.

### 3.3. Parameter Learning for ANN Models

It is important to mention that there were empirical attempts to define the number of neurons in the hidden layer and transfer functions in the hidden and output layers. Empirical attempts considering 2, 4, 8, 16, 32, 64, and 128 neurons in the hidden layer were done and alternating the transfer function resulted in a small variation in training, validating, and testing MSE of 0.5%. Therefore, it was decided to generate simpler models according to the parameters explained in Table 4.

It is important to mention that the models were generated using MATLAB^®^ version R2018a (The MathWorks Inc.: Natick, MA, USA) on a computer equipped with a processor by Intel^®^ Core™ i7-3537U, CPU 2.00 GHz, 8 GB RAM, SSD (Solid State Disk).

## 4. Results and Discussion

**Figure 11:**
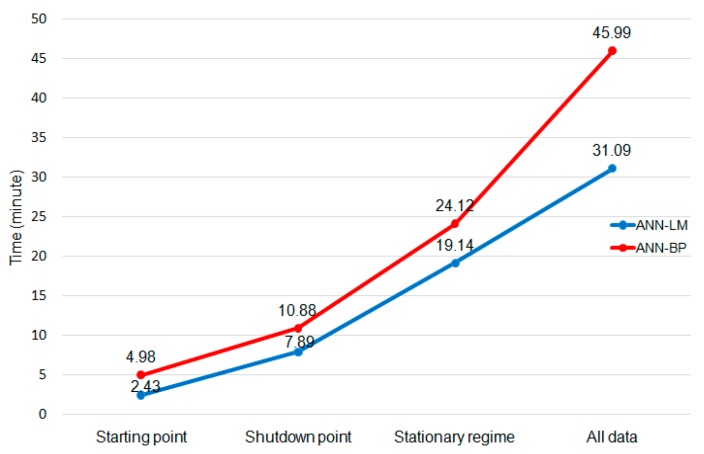
Time spent on ANN- Levenberg–Marquardt (LM) and ANN-back propagation (BP) experiments.

After running the experiments, this section shows and discusses the results. Figure 11 shows the time spent in each set of experiments by lifespan division and the training algorithm. Once there were 32 different sections, three different outputs and ten experiments were done, so each point represents the training of 960 different models. All experiments consumed over two and a half hours in total, where the LM algorithm was almost twice as fast as the BP.

**Figure 12:**
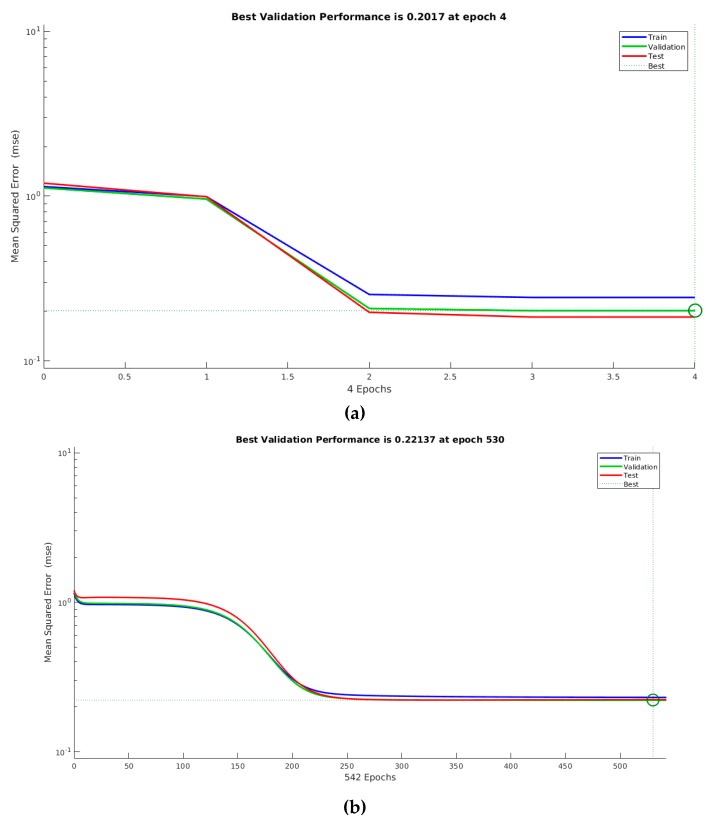
Examples of the evolution of training, validating and testing of neural networks creation process for TMP output: (**a**) LM algorithm; and (**b**) BP algorithm.

Figure 12 exemplifies the evolution of training, validating and testing of neural networks creation process for TMP output, considering starting point data. It is possible to verify LM converges faster and it is more accurate than BP. This same behavior was identified for the other outputs and lifespan divisions.

Since the reduction pot always operates with the closed loop control, the available data are closed loop. In other words, the estimation of the variables made by the soft sensors is in a closed loop. Thus, the estimates obtained show bias deviations and inherent error in the frequency domain [72,73,74,75,76]. Since the reduction pot cannot operate in an open loop, these errors will be inherent in the estimates obtained, but are sufficiently useful for control [73,76]. Therefore, it is possible that data are affected by the change of the controller transfer function.

**Figure 13:**
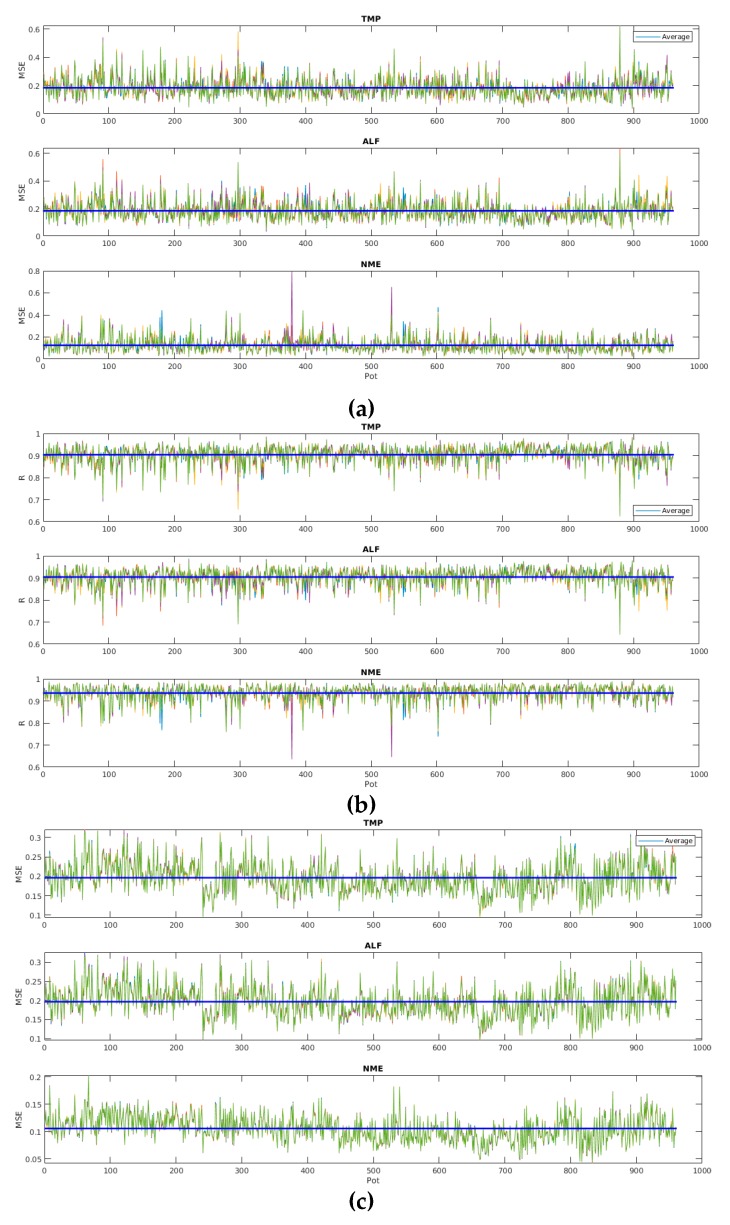

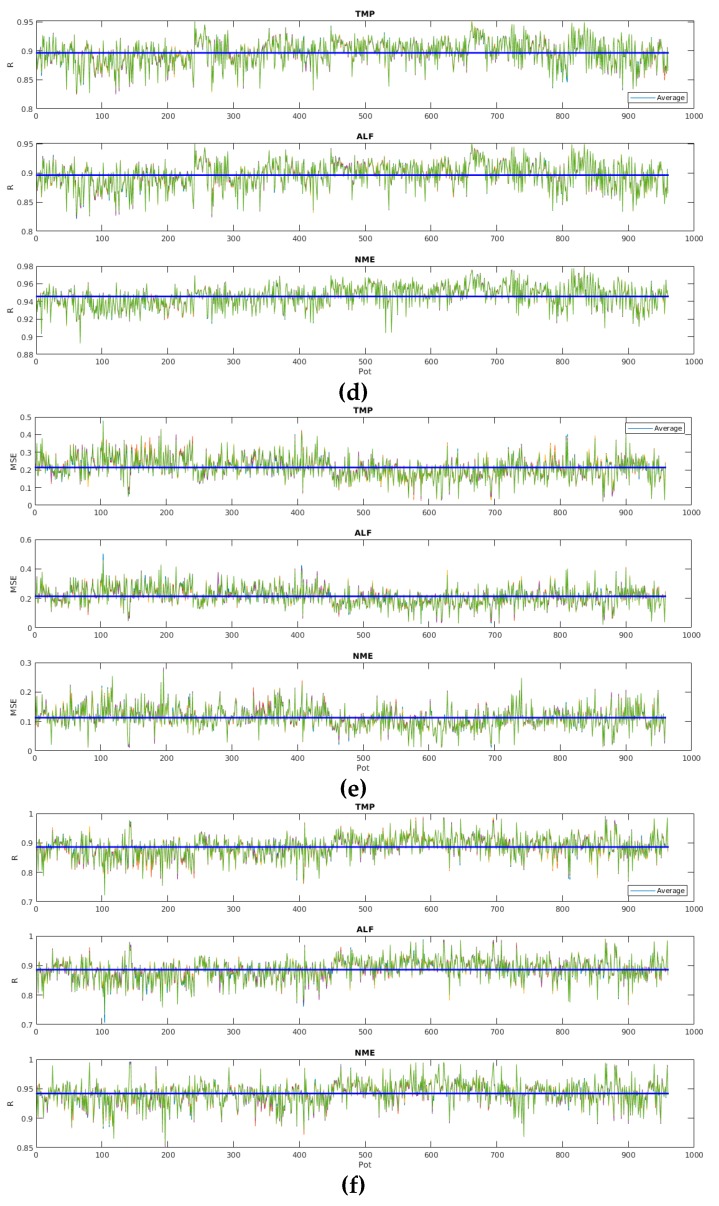
Mean squared error (MSE) and R values of ANN-LM based models considering the 2880 models: (**a**) MSE for starting point; (**b**) R for starting point; (**c**) MSE for stationary regime; (**d**) R for stationary regime; (**e**) MSE for shutdown point; and (**f**) R for shutdown point.

Figure 13 shows MSE and R values for 2880 models considering all pots in starting, stationary and shutdown phases, ANN-LM, the three output variables, and normalized data. Most models present low MSE values and high R values (the blue line is the average). Therefore, the contribution is to prove that the modeling strategy described worked properly.

**Figure 14:**
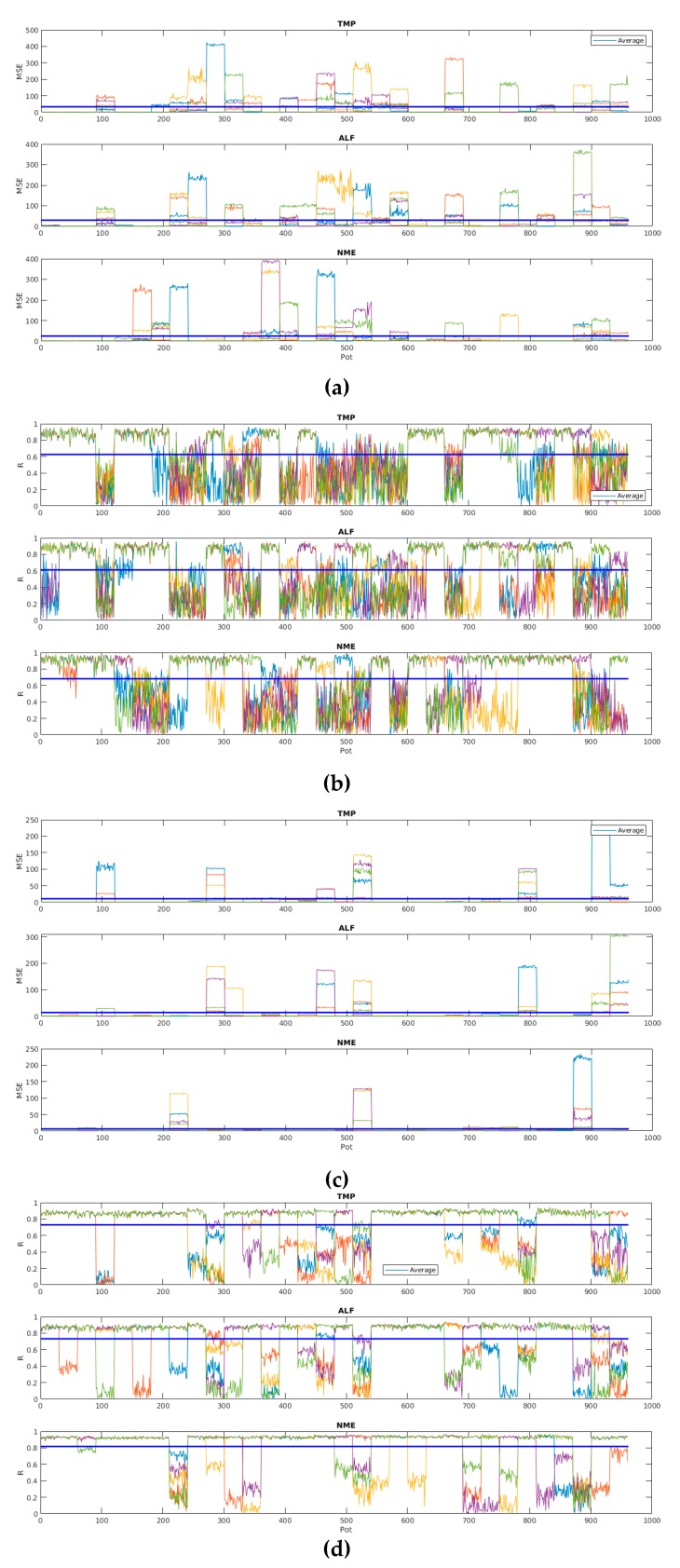

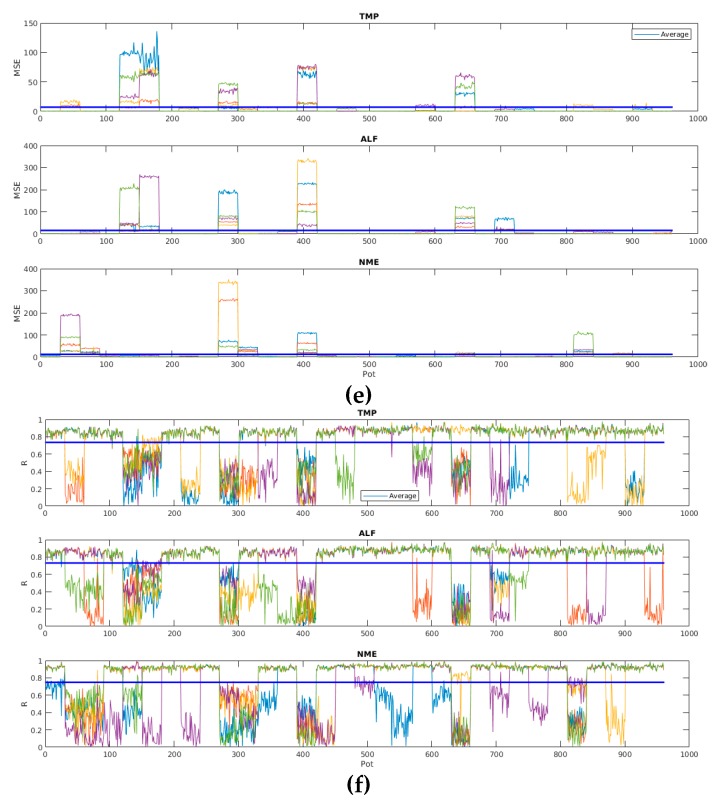
MSE and R values of ANN-BP-based models considering the 2880 models: (**a**) MSE for starting point; (**b**) R for starting point; (**c**) MSE for stationary regime; (**d**) R for stationary regime; (**e**) MSE for shutdown point; and (**f**) R for shutdown point.

Figure 14 shows MSE and R values for the other 2880 models, considering all the characteristics and pots previously mentioned, but the ANN-BP training algorithm. It is noted that MSE and R values were bigger on average and had more variants than those of ANN-LM. It is interesting to note high variance in the results of each section.

**Figure 15:**
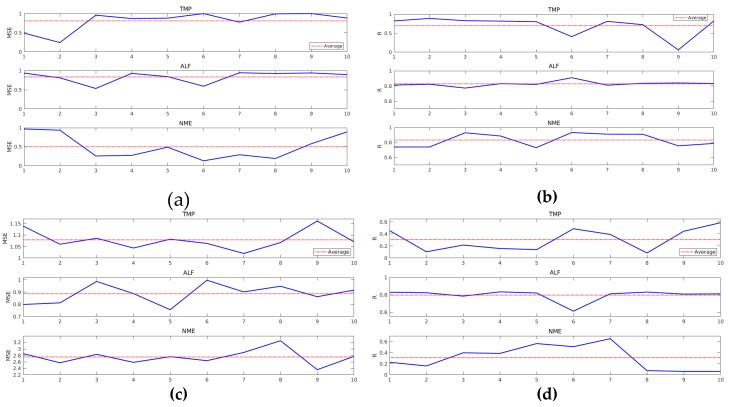
MSE and R values of ANN-LM- and ANN-BP-based models considering models created by all data: (**a**) MSE for ANN-LM; (**b**) R for ANN-BP; (**c**) MSE for ANN-BP; and (**d**) R for ANN-BP.

Figure 15 shows MSE and R values for models created by all data for ANN-LM and ANN-BP. It was possible to verify higher MSE and lower R (on average) when compared to previous models.

Table 5 outlines MSE and R average (avg) and standard deviation (std) global values, besides minimum and maximum MSE and R values in all 5760 models. It is possible to verify that the LM algorithm generates more accurate models in all cases. The quality of the estimation is much better when LM is considered; it may be check analyzing the high values of BP’s avg and std.

Comparative graphs between target values and estimated by the models were generated after the creation of estimating models and selection of the best ones. Once there were 32 models for three different lifespan divisions, models based on all data, three outputs (TMP, ALF, and NME), and two ANN learning algorithms, then it was necessary to select only one pot to visualize this similarity (pot 5).

**Figure 16:**
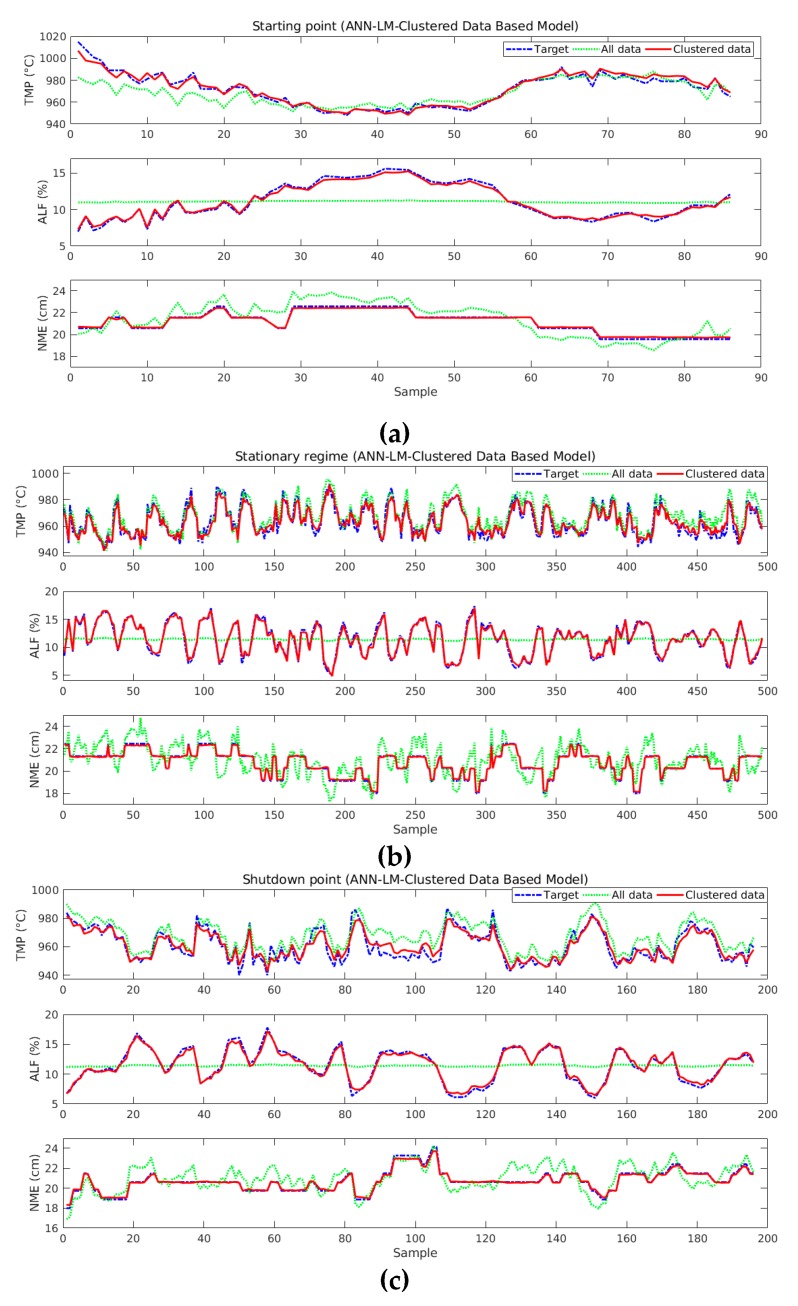
Comparison between target and estimated values for ANN-LM-based models and by clustered and all data: (**a**) starting point; (**b**) stationary regime; and (**c**) shutdown point.

Figure 16 displays comparisons for ANN-LM-based models considering non-standardized data. It verified that the models based on lifespan division (red line) estimate very well the dynamics of the process for all output variables. Models based on all data had not learned to estimate the values (green line), especially the ALF output. Next to the graphs, there were the respective MSE and R values.

**Figure 17:**
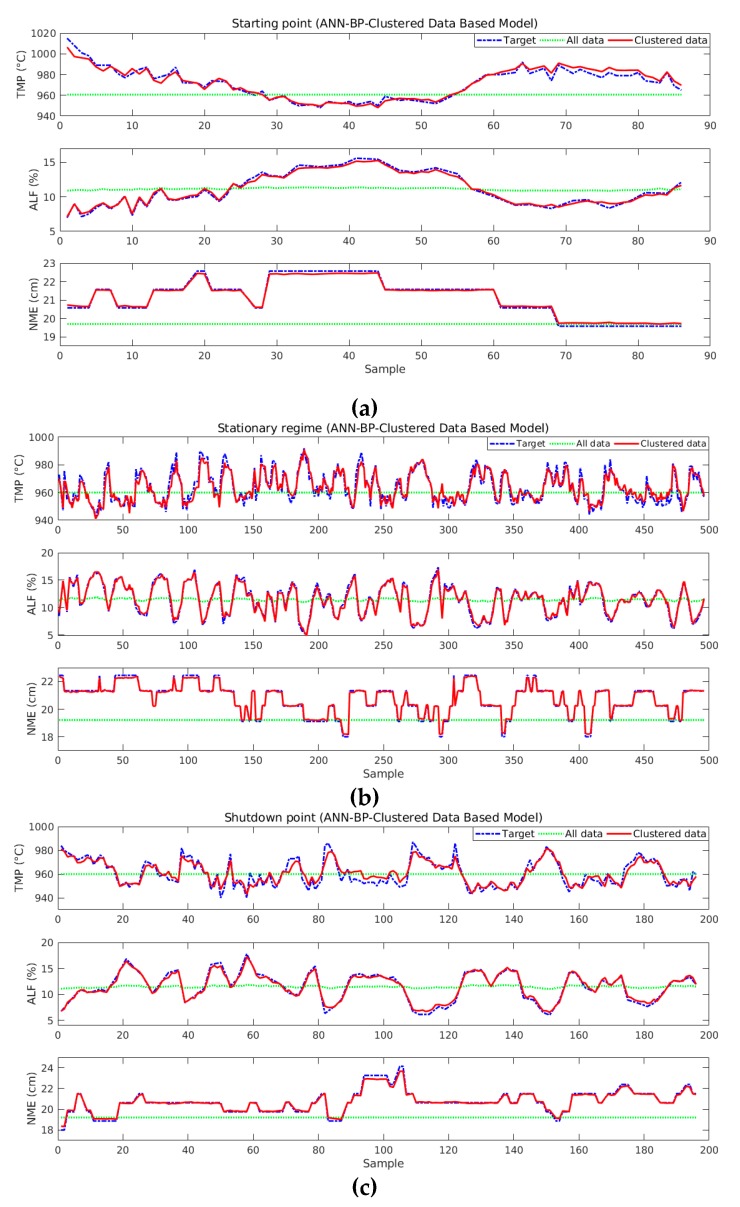
Comparison between target and estimated values for ANN-BP-based models and by lifespan division: (**a**) starting point; (**b**) stationary regime; and (**c**) shutdown point.

Figure 17 shows comparisons for ANN-BP-based models. Estimated values also follow target values, but the accuracy is lower than the ANN-LM-based models for the most variables. When models based on all data are analyzed, it is possible to verify that they have not learned using the neural network parameters cited above.

Table 6 displays the MSE and R values for comparisons between target and estimated values for ANN-LM, ANN-BP-based models and by clustered and all data plotted on the graphs in Figure 16 and Figure 17. It proves the advantage of using the proposed method. It is important to remember that data used to perform these comparisons were not used in the neural net creation process.

**Figure 18:**
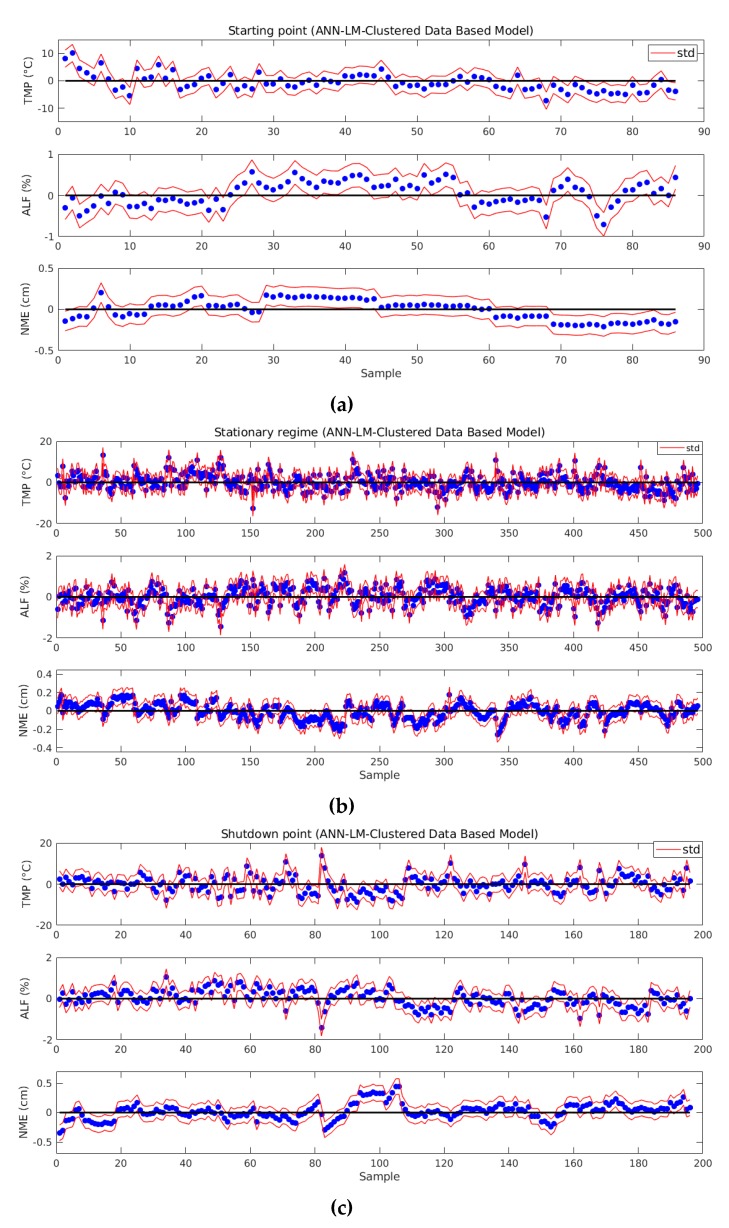
Residual plots: (**a**) starting point; (**b**) stationary regime; and (**c**) shutdown point.

Another results evaluation was performed analyzing residual plot in all phases, considering the best clustered based model. Figure 18 shows that the most TMP points are between −5 °C and 5 °C, the most ALF points are between −1% and 1%, and NME points are between −0.5 cm and 0.5 cm. These error variances are perfectly acceptable by process engineer. Red lines display the std ranges.

## 5. Conclusions

In this work, the results of an innovative approach to create soft sensors to estimate TMP, ALF, and NME variables of primary Al production were presented. After testing different neural net topologies and considering two different training algorithms, training and testing 5940 different models, the best model of each output variable was selected and it was possible to ensure that these models generate high generalization power and very small errors that are fully tolerated by process engineers. In all cases, models based on section clustering and lifespan division performed more accurate estimates compared to models that do not use clustering. LM has helped to create neural networks more accurate than the BP algorithm. Besides, LM is faster for training the models.

TMP, ALF, and NME variables are the most important to control the proper functioning of the pots. The lifespan and section dataset clustering contributed to creating more specialized models in the behaviors of the respective clusters of pots, reducing errors and increasing the precision of the estimating soft sensors. ANNs have been chosen because they can generate models with a high power of generalization and they have the capability to learn the nonlinearity of the process using experimental plant data.

MATLAB^®^ was used to develop the models, but a computer system will be created to implement the integration of soft sensors with data acquired in real time, making it possible for engineers to virtually estimate the behavior of the pots, rather than make manual or laboratory measurements. It is planned to use these soft sensors to control the pots.

## Figures and Tables

**Table 1:** All variables available in the database.

Abbreviation	Complete Name	Unit
%CaO	Calcium Oxide Percentage	%
%Fe_2_O_3_	Iron Oxide Percentage	%
%MnO	Manganese Dioxide Percentage	%
%Na_2_O	Sodium Oxide Percentage	%
%P_2_O_5_	Phosphorus Pentoxide Percentage	%
%SiO_2_	Silicon Oxide Percentage	%
%TiO_2_	Titanium Dioxide Percentage	%
%V_2_O_5_	Vanadium Pentoxide Percentage	%
%ZnO	Zinc Oxide Percentage	%
<325 m	<325 Mesh	%
>100 m	>100 Mesh	%
>200 m	>200 Mesh	%
CR	Friction Index	%
CRF	Thin Crust	%
DA	Apparent Density	g/cm^3^
LOI1	Loss on ignition (300–1000 °C)	%
LOI2	Loss on ignition (110–1000 °C)	%
LOI3	Loss on ignition (110–300 °C)	%
SE	Specific Surface	m^2^/g
%FE	Iron Content in Metal	ppm
%Ga	Gallium Content	%
%Mn	Manganese Content	%
%Na	Sodium Content in Metal	%
%Ni	Nickel Content	%
%P	Metal Phosphorus Content	ppm
%SI	Silicon Content in Metal	ppm
%TBase	Percentage of Time on Base Feed	%
%TChk	Check Feed Time Percentage	%
%TInic	Percentage of Initial Feeding Time	%
%TOthers	Percentage of Time Other Feeding Modes	%
%TOV	Percentage of Feeding Over Time	%
%TUN	Percentage of Feeding Time Under	%
%V_	Vanadium Content	%
A%1	Feeding (Al_2_O_3_)	%
ALF	Aluminum Fluoride (% in Bath)	%
ALF3A	Amount of AlF3 Added	kg/Misc
ALF3AB	AlF3–Base Addition–Total	kg/Misc
ALF3ABF	AlF3–Base Addition–ABF	kg/t Al
ALF3ABFC	AlF3–Base Addition–Factor C	kg/t Al
ALF3ABN	AlF3–Base Addition–Na_2_O	kg/t Al
ALF3ABT	AlF3–Base Addition–Total	kg/Misc
ALF3ABV	AlF3–Base Addition–Life	kg/Misc
ALF3Ac	Amount of AlF3 Added–Correction	kg/Misc
ALF3AE	ALF3A–Extra Addition	kg/Misc
ALF3Ah	Amount of AlF3 Added–Historic	kg/Misc
ALF3Am	Amount of AlF3 Added–Maintenance	kg/Misc
ALF3AR	AlF3 Deviation Reference	kg/Misc
ALF3ARB	ALF3A–[Real–Base]	kg/Misc
ALF3AS	AlF3–Hopper Balance Correction	kg/Misc
ALF3At	Amount of AlF3 Added–Trend	kg/Misc
ALF3ATS	Hopper Balance	kg/Misc
ALF3ATSAc	Accumulated Hopper Balance	kg/Misc
ALF3CA	AlF3–% AlF3 Correction	kg/Misc
ALF3CM	AlF3 Quantity–Manual Correction	kg/Misc
ALF3CT	AlF3–Temperature Correction	kg/Misc
ALF3DA	AlF3 Added–Cumulative Deviation	kg
ALF3DALI	AlF3–Accumulated Deviation–Lower Limit	kg
ALF3DALS	AlF3–Accumulated Deviation–Upper Limit	kg
ALF3LC	AlF3–Limit Check Correction	kg/Misc
ALFca	Aluminum Fluoride for CA	%
ALFcalc	Calculated Aluminum Fluoride	%
ALM	Feeder	Kg
CAF	Calcium Fluoride (% in Bath)	%
CAF2A	Amount of CaF_2_ Added	kg
CAF2CM	CaF_2_ Quantity–Manual Correction	kg
CAN	Anode Coverage	cm
CE	Specific Energy Consumption	kWh/kg Al
CoLiq	Liquid Column	cm
CQB-Efetiv	Chemical Bath Control—Effectiveness	%
DeltaR	Resistance Delta	uOhm
DeltaT	Super Heat	°C
DeltaT1	Super Heat	°C
DeltaTM	Super Heat Measured	°C
DeltRCI	DeltaR–Instability Calculation	uOhm
DesAnodCAR	Anode Descent in CAR	un
DesAutAnod	Automatic Anode Descent	un
DifNME	Metal Level (Real-Set)	cm
DifRMR	Rreal-Rset	uOhm
DifRSO	Rtarget-Rset	uOhm
DRPTro	Post-Trade Resistance Delta	uOhm
EaEnergL	Anode Effect (AE)–Net Energy	Kwh/EA
EAN	Unscheduled Anode Effect	EA/d
EAP	Scheduled Anode Effect	ea/d
EaDurPol	AE–Polarization Duration	seg/Ea
EaDurPolTot	AE–Total Duration of Polarization	seg/F/Day
EaVBruta	AE–Gross Voltage	V/Ea
EaVLiq	AE–Liquid Voltage	V/Ea
EaVMax	AE–Maximum Voltage	V
EaVPol	AE–Voltage Polarization	V/Ea
ECO	Current Efficiency	%
FAB	AlF3 Base Addition	kg/Misc
FARB	Addition (Real + Extra − Base)	kg/Misc
IMx	Current Intensity	kA
IncCTAlim	Increment–CTFeed	uOhm
IncCTOsc	Increment–CTOsc	uOhm
IncOp	Increment–Operation	uOhm
IncOs	Increment–Oscillation	uOhm
IncTm	Increment–Temperature	uOhm
IncTr	Increment–Anode Exchange	uOhm
Na	Sodium Content in Metal (PPM)	ppm
NA2CO3A	Added Amount of Na_2_CO_3_	kg
NA2CO3CM	Na_2_CO_3_ Quantity–Manual Correction	kg
NBA	Bath Level	cm
NBAA	Bath Addition	Kg
NBAc	Bath Control	Kg
NBAR	Bath Removal	Kg
NCicSEA	SEA Cycle Number	Ciclos/SEA
NEA	Total Anode Effect	ea/d
NEARecorr	Total Recurrent Anode Effect	EA/d
NME	Metal Level	cm
NOV	Number of Overs	un
NSA	Number of Feed Shots	un
NTR	Number of Tracks	-
NumOverUnder	Number of Overs Followed by Unders	un
PAN	Anodic Loss	uOhm
PCA	Cathodic Loss	mV
PCO	Cathodic Loss (uOhms)	mOhm
PHV	Loss Rod Beam	uOhm
PreEA	Anode Pre-Effect	ea/d
PrvEA	Anode Effect Prediction	ea/d
PUR	Metal Purity (% Al)	%
QALr	Feed Quantity (Real)	kg
QALt	Feed Quantity (Theoretical)	kg
QME	Amount of Flushed Metal (Real)	ton
RMR	Real Resistance	uOhm
RS	Resistance Setpoint	uOhm
RSO	Target Resistance	uOhm
SetNBA	Bath Level Setpoint	cm
SetNME	Metal Level Setpoint	cm
SILO	Alf3 Silo Filling Control	-
SIM	Impossible Anode Effect Suppression	%
SIMTot	Impossible Total Anode Effect Suppression	%
SPEA	Anode Pre-Suppression	ea/d
SPEAIM	Impossible Anode Pre-Effect Suppression	%
SubAnodCAR	CAR Anode Rise	un
SubAutAnod	Automatic Anode Rise	un
SWF	Strong Oscillation	%
SWT	Total Oscillation	%
TAS	Suspended Feed Time	min
TC1	Check Time	min
TEA	Anode Effect Time	min
TMP	Bath Temperature	°C
TMPcat	CA Bath Temperature	°C
TMPLI	Bath Temperature–Lower Limit	°C
TMPLiq	Liquid Temperature	°C
TMPLS	Bath Temperature–Upper Limit	°C
TMT	Track Time	min
TOV	Over Time	min
TUN	Under Time	min
VIDA	Pot Life	days
WF	Real Consumption of Oven	kW
WFA	Oven Target Consumption	kW
AF	Fresh Alum Silo Level	%
af%F	Adsorbed Fluoride (Fluorinated Alumina)	%
af%F(Cor)	Corrected plant fluoridation	%
af%Na2O	Sodium Oxide (Fluorinated Alumina)	%
af%UM	Moisture (Fluorinated Alumina)	%
Af < 325 m	<325 Mesh (Fluorinated Alumina)	%
Af < 400 m	<400 Mesh (Fluorinated Alumina)	%
Af > 100 m	>100 Mesh (Fluorinated Alumina)	%
Af > 200 m	>200 Mesh (Fluorinated Alumina)	%
afDA	Apparent Density (Fluorinated Alumina)	g/cm^3^
afLOI1	L.O.I. (110–300 °C; AF)	%
AluT	Transported Alumina	T
Na2Odif	Sodium Oxide (Fluorinated Alumina–Virgin)	%
SPVZ	Fresh Alumina Flow Setpoint	T/h
VZ	Fresh Alumina Flow	T/h
af%UMx	Moisture (Fluorinated Alumina)	%
ALF LI	Lower Limit ALF	%
ALF LS	ALF Upper Limit	%
IA	Target Current	kA
IM	Current Intensity	kA
IMBB	Booster Current Intensity	kA
IMC	Current Intensity (Pot)	kA
IMRB	Current Intensity	kA
VL	Line Voltage	V
WL	Actual Line Consumption	MW
ECp	Predicted Current Efficiency	%
ECr	Real Current Efficiency	%
PRODReal	Real Production	t

**Table 2:** Variables used for the modeling.

ID	Type	Variable	Abbreviation	Unit	Delay	R w/TMP	R w/ALF	R w/NME
1	Input	Gross Voltage	VMR-1	V	1-step	−0.49	0.43	0.30
2		Gross Resistance	RMR-1	uOhm		−0.48	0.41	0.24
3		Bath Level	NBA-1	cm		0.58	−0.41	−0.69
4		Calcium Fluoride (% in the Bath)	CAF-1	%		−0.53	−0.49	0.37
5		Percentage of Sodium Oxide	PNA2O-1	%		−0.52	−0.67	0.31
6		Percent of Calcium Oxide	PCAO-1	%		−0.57	0.72	0.32
7		Amount of AlF3 Added	ALF3A-1	kg/misc		0.40	−0.46	−0.30
8		Amount Fed (Real)	QALR-1	kg		−0.35	0.32	0.52
9		Temperature	TMP-1	°C		0.88	−0.79	0.32
10		Aluminum Fluoride (% in the Bath)	ALF-1	%		−0.78	0.94	0.25
11		Metal Level	NME-1	cm		−0.41	0.34	0.94
12	Output	Temperature	TMP	°C		-	-	-
13		Aluminum Fluoride (% in the Bath)	ALF	%	-	-	-	-
14		Metal Level	NME	cm		-	-	-

**Table 3:** Complete modeling process.

Lifespan Division	Training Algorithm	Number of Models
Starting point	ANN-LM	32 sections × 3 outputs = 96 All dataset × 3 outputs = 3
	ANN-BP	32 sections × 3 outputs = 96 All dataset × 3 outputs = 3
Stationary regime	ANN-LM	32 sections × 3 outputs = 96 All dataset × 3 outputs = 3
	ANN-BP	32 sections × 3 outputs = 96 All dataset × 3 outputs = 3
Shutdown point	ANN-LM	32 sections × 3 outputs = 96 All dataset × 3 outputs = 3
	ANN-BP	32 sections × 3 outputs = 96 All dataset × 3 outputs = 3
	TOTAL	576 models (clustered data) 18 models (all dataset) 594 models

**Table 4:** Artificial neural network (ANN) model details.

Parameter	Value	Justification
Number of hidden layers	1	Empirical attempts.
Number of neurons in the hidden layer	2	
Transfer function in the hidden layer	Symmetric Sigmoid	
Transfer function in the output layer	Linear	
Learning algorithms	LM	To build models faster, because this algorithm considers an approximation of Newton’s method, which uses an array of second-order derivatives and a first-order derivative matrix (Jacobian matrix). On the other hand, it uses more memory to calculate optimal weights [76,77].
	BP	To create models based on the most traditional learning algorithm: descendent gradient. It is slower than LM, but it uses less memory [78,79].

**Table 5:** Compendium of MSE and R global values considering all models.

Lifespan Division	ANN Training Algorithm	Output Variable	MSE_global_	R_global_	MIN and MAX MSE	MIN and MAX R
Starting point	LM	TMP	avg: 0.182 std: 0.001	avg: 0.903 std: 0.0006	0.031; 0.639	0.623; 0.986
		ALF	avg: 0.124 std: 0.002	avg: 0.935 std: 0.0009	0.015; 0.899	0.568; 0.993
		NME	avg: 0.110 std: 0.0008	avg: 0.927 std: 0.0005	0.001; 0.496	0.727; 0.997
	BP	TMP	avg: 31.833 std: 13.102	avg: 0.618 std: 0.013	0.053; 424.58	2.5 × 10^−5^; 0.973
		ALF	avg: 28.133 std: 22.021	avg: 0.675 std: 0.017	0.029; 460.52	0.0002; 0.988
		NME	avg: 69.322 std: 23.053	avg: 0.333 std: 0.011	0.005; 668.16	8.6 × 10^−6^; 0.971
Stationary regime	LM	TMP	avg: 0.196 std: 0.0001	avg: 0.896 std: 8.5 × 10^−5^	0.093; 0.326	0.821; 0.952
		ALF	avg: 0.105 std: 5.5 × 10^−5^	avg: 0.945 std: 3.0 × 10^−5^	0.041; 0.205	0.891; 0.979
		NME	avg: 0.129 std: 7.9 × 10^−5^	avg: 0.932 std: 3.6 × 10^−5^	0.002; 0.299	0.839; 0.982
	BP	TMP	avg: 12.45 std: 12.84	avg: 0.731 std: 0.042	0.109; 310.31	0.0002; 0.943
		ALF	avg: 4.84 std: 11.96	avg: 0.817 std: 0.041	0.057; 234.28	0.0005; 0.970
		NME	avg: 41.15 std: 39.82	avg: 0.526 std: 0.015	0.015; 946.94	7.7 × 10^−5^; 0.972
Shutdown point	LM	TMP	avg: 0.213 std: 0.0004	avg: 0.886 std: 0.0003	0.018; 0.503	0.705; 0.991
		ALF	avg: 0.112 std: 0.0003	avg: 0.941 std: 0.0001	0.010; 0.283	0.850; 0.996
		NME	avg: 0.184 std: 0.0003	avg: 0.897 std: 0.0001	0.001; 0.462	0.742; 0.998
	BP	TMP	avg: 11.36 std: 17.93	avg: 0.730 std: 0.033	0.047; 342.54	0.0008; 0.976
		ALF	avg: 14.34 std: 27.38	avg: 0.742 std: 0.025	0.017; 634.69	5.1 × 10^−5^; 0.991
		NME	avg: 11.36 std: 17.93	avg: 0.581 std: 0.015	0.006; 725.00	2.3 × 10^−5^; 0.990
All data	LM	TMP	avg: 0.80 std: 0.25	avg: 0.70 std: 0.26	0.241; 0.990	0.061; 0.890
		ALF	avg: 0.83 std: 0.15	avg: 0.82 std: 0.03	0.534; 0.945	0.772; 0.909
		NME	avg: 0.50 std: 0.32	avg: 0.83 std: 0.08	0.131; 0.969	0.730; 0.932
	BP	TMP	avg: 1.07 std: 0.04	avg: 0.30 std: 0.18	1.020; 1.160	0.084; 0.585
		ALF	avg: 0.88 std: 0.08	avg: 0.79 std: 0.06	0.756; 0.996	0.612; 0.833
		NME	avg: 2.75 std: 0.23	avg: 0.30 std: 0.22	2.359; 3.252	0.061; 0.649

**Table 6:** MSE and R values by training algorithm, lifespan division, and data type.

ANN Training Algorithm	Lifespan Division	Data Type	MSE	R
LM	Starting point	Clustered	TMP: 9.939 ALF: 0.083 NME: 0.014	TMP: 0.977 ALF: 0.996 NME: 0.999
		All data	TMP: 73.18 ALF: 5.39 NME: 0.54	TMP: 0.809 ALF: 0.867 NME: 0.913
	Stationary regime	Clustered	TMP: 14.37 ALF: 0.179 NME: 0.007	TMP: 0.941 ALF: 0.989 NME: 0.999
		All data	TMP: 53.12 ALF: 6.92 NME: 1.00	TMP: 0.874 ALF: 0.733 NME:0.905
	Shutdown point	Clustered	TMP: 15.669 ALF: 0.1652 NME: 0.018	TMP: 0.940 ALF: 0.991 NME: 0.998
		All data	TMP: 48.58 ALF: 6.92 NME: 0.83	TMP: 0.888 ALF: 0.757 NME: 0.839
BP	Starting point	Clustered	TMP: 10.96 ALF: 0.077 NME: 0.012	TMP: 0.975 ALF: 0.996 NME: 0.999
		All data	TMP: 139.13 ALF: 5.19 NME: 3.17	TMP: −0.760 ALF: 0.779 NME: 0.818
	Stationary regime	Clustered	TMP: 14.06 ALF: 0.177 NME: 0.010	TMP: 0.942 ALF: 0.989 NME: 0.999
		All data	TMP: 141.94 ALF: 6.57 NME: 3.51	TMP: −0.663 ALF: 0.782 NME:0.775
	Shutdown point	Clustered	TMP: 16.624 ALF: 0.158 NME: 0.020	TMP: 0.935 ALF: 0.992 NME: 0.998
		All data	TMP: 137.31 ALF: 6.60 NME: 3.53	TMP: −0.542 ALF: 0.863 NME: 0.831
